# *Lecanorchis tabugawaensis* (Orchidaceae, Vanilloideae), a new mycoheterotrophic plant from Yakushima Island, Japan

**DOI:** 10.3897/phytokeys.73.10019

**Published:** 2016-11-03

**Authors:** Kenji Suetsugu, Hirokazu Fukunaga

**Affiliations:** 1 Department of Biology, Graduate School of Science, Kobe University, 1-1 Rokkodai, Nada-ku, Kobe, 657-8501, Japan; 2 Tokushima-cho 3-35, Tokushima City, Tokushima, Japan

**Keywords:** IUCN conservation status, mycoheterotrophy, new species, reproductive biology, taxonomy

## Abstract

A new species, *Lecanorchis tabugawaensis* Suetsugu & Fukunaga, **sp. nov.** from Yakushima Island, Kagoshima Prefecture, Japan, is described and illustrated. *Lecanorchis tabugawaensis* is similar to *Lecanorchis taiwaniana*, but it is easily distinguished by the straight column, the glabrous status of the base of the column, the almost entire and narrow labellum morphology, the shorter part of the column fused with the labellum and the glabrous status of the apical part of the adaxial labellum surface. The new species appears to be restricted to two locations, each consisting of only dozens of mature individuals, and is assessed as Critically Endangered [CR D1] according to IUCN Red List Categories and Criteria.

## Introduction

The genus *Lecanorchis* Blume comprises a group of mycoheterotrophic plants with an erect stem which may be either branched or unbranched (Hashimoto 1990; Seidenfaden 1978; Szlachetko and Mytnik 2000; Hsu and Chung 2010). A key characteristic of the species of *Lecanorchis* is the presence of a calyculus, a cup-like structure between the base of the perianth and the apex of the ovary (Cameron 2003; Hashimoto 1990; Sawa et al. 2006). There are over thirty species and/or varieties in the genus *Lecanorchis* extending across a large area that includes Thailand, Malaysia, Indonesia, Vietnam, the Philippines, Taiwan, Japan, and New Guinea (Hashimoto 1990; Seidenfaden 1978; Su 2000; Szlachetko and Mytnik 2000; Cameron 2003; Averyanov 2011). Precise *Lecanorchis* species identification is often difficult due to close similarities in morphology and the short durations of flowering periods (Hashimoto 1990; Averyanov 2005; Suddee and Pedersen 2011; Tsukaya and Okada 2013; Suetsugu et al. 2016a). This challenge remains due to the difficulty of identification at the fruiting stage even though the *Lecanorchis* species maintains withered plants above ground levels for longer periods compared to other mycoheterotrophic species, and the fruiting plants can be easily found in the forests. Furthermore, detailed descriptions for some species remain lacking, particularly for those first described decades ago (Suetsugu et al. 2016a). Given such difficulties in precise identification, adequate taxonomic studies of this genus have not been conducted.

Nevertheless, Japan is known for its great diversity of *Lecanorchis*, harboring ca. ten species and/or varieties (Hashimoto 1990). In fact, the flora of Japan is particularly rich in mycoheterotrophic plants, and recent botanical surveys of the mycoheterotrophic plants in Japan resulted in the discovery of several new distributional records and new taxa of mycoheterotrophic species (Sawa et al. 2006; Fukunaga et al. 2008; 2016; Yagame et al. 2008; Yahara and Tsukaya 2008; Ohashi et al. 2008; Suetsugu et al. 2012, 2013, 2014a, 2016a, b; Suetsugu 2012, 2013a, 2014, 2015a, 2016a, b, c, d; Suetsugu and Ishida 2011; Suetsugu and Yagame 2014). Of particular interest are the lowland evergreen forests of Yakushima Island, which are known to be a hotspot for endemic taxa, including the mycoheterotrophic plants such as *Oxygyne yamashitae* Yahara & Tsukaya and *Sciaphila yakushimensis* Suetsugu, Tsukaya & H. Ohashi. A detailed botanical survey of Yakushima Island would, therefore, hopefully provide more precise data regarding diversity of the species and distribution of the mycoheterotrophs (Suetsugu 2015; Suetsugu et al. 2016b). As anticipated, a new *Lecanorchis* species, with significantly different floral morphology compared to other known species, was discovered in the lowland evergreen forest of Yakushima.

## Taxonomy

### Lecanorchis tabugawaensis

Taxon classificationPlantaeAsparagalesOrchidaceae

Suetsugu & Fukunaga sp. nov.

urn:lsid:ipni.org:names:77158316-1

Figs 1 , 2 , 3

#### Diagnosis.

*Lecanorchis tabugawaensis* differs from its close relative *Lecanorchis taiwaniana* in having a straight column, a narrow and almost entire labellum and the glabrous apical part of the adaxial labellum surface.

#### Type.

JAPAN. Kyushu: Kagoshima Pref., Yakushima Island, Yakushima Town, Koseda, along Tabu River, alt. 170m, 16 July 2015, *H. Yamashita s.n.* (holotype KYO; isotype OSA).

#### Additional specimen examined.

JAPAN. Kyushu: Kagoshima Pref., Yakushima Island, Yakushima Town, Koseda, along Tabu River, alt. 170m, 9 October 2015, K. Suetsugu *s.n.* (OSA).

#### Description.

Terrestrial, mycoheterotrophic herb. Inflorescence 15–45 cm tall, unbranched or branched at lower half, yellowish white at flowering, brownish black at fruiting, glabrous, ca. 1.0 mm in diam., with membranaceous scale-like sheaths. Rachis 6–15 cm, 4–15 flowered, internodes 5–15 mm apart. Floral bracts deltoid, ca. 2.0 mm long, ca. 1.0 mm wide. Pedicellate ovary ascending, 15–20 mm long. Sepals and lateral petals widely spreading, ca. 2.5 cm in diameter. Sepals yellowish white, linear, slightly narrower in lower half, 14–17 mm long, ca. 1.8–2.5 mm wide, apex obtuse, 3-nerved. Petals yellowish white, linear, slightly oblique, 14–17 mm long, ca. 2.0–2.5 mm wide, apex obtuse, 3-nerved. Labellum white tinged with purple toward apex, glabrous, 14–15 mm long, ca. 5 mm wide when flattened, entire. Column 12–13 mm long, straight, fused with labellum for about 2/5–1/2 its length, glabrous; anther whitish, ca. 1.5 mm wide. Capsule 20-30 mm long, bright brown, ascending at 20-45° angle from axis.

**Figure 1.**
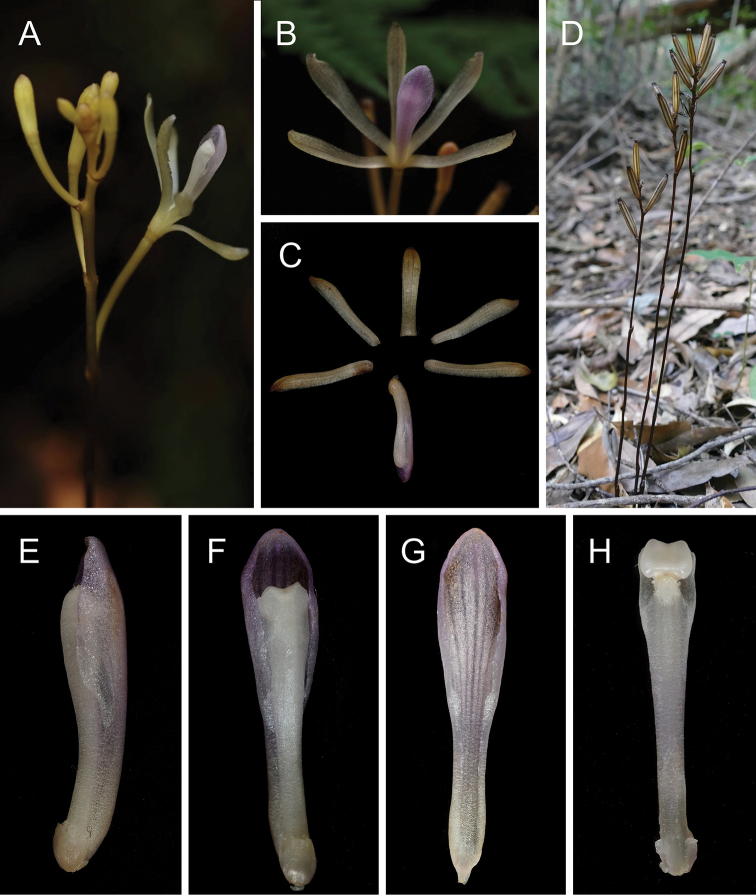
Photographs of *Lecanorchis tabugawaensis* in Taiwan. **A** Flowering habit **B–C** Flower **D** Fruiting habit **E–F** Lip and column **G** Lip **H** Column. Photos by Hiroaki Yamashita (**A–B**), Takuto Shitara (**C, E–H**) and Kenji Suetsugu (**D**).

**Figure 2.**
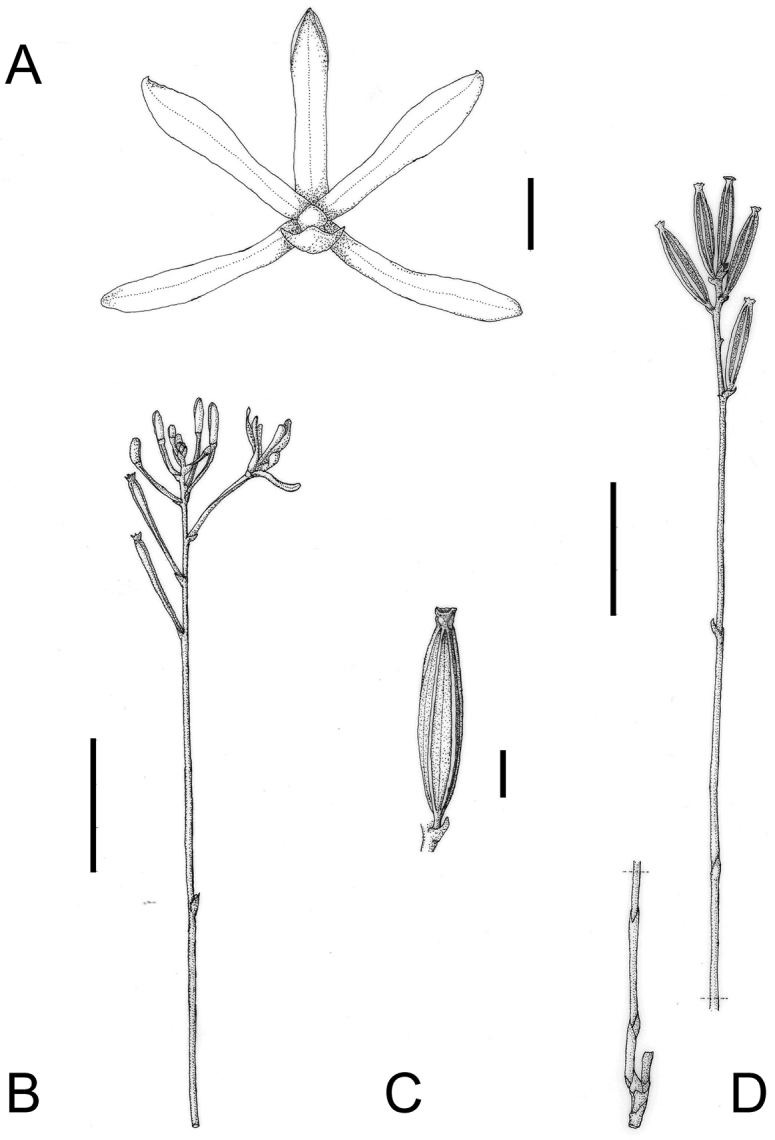
*Lecanorchis tabugawaensis*. **A** Flower **B** Flowering habit **C** Fruit **D** Fruiting habit. Bars: 5 mm (**A, C**); 3 cm (**D**). Line drawings by Kumi Hamasaki.

**Figure 3.**
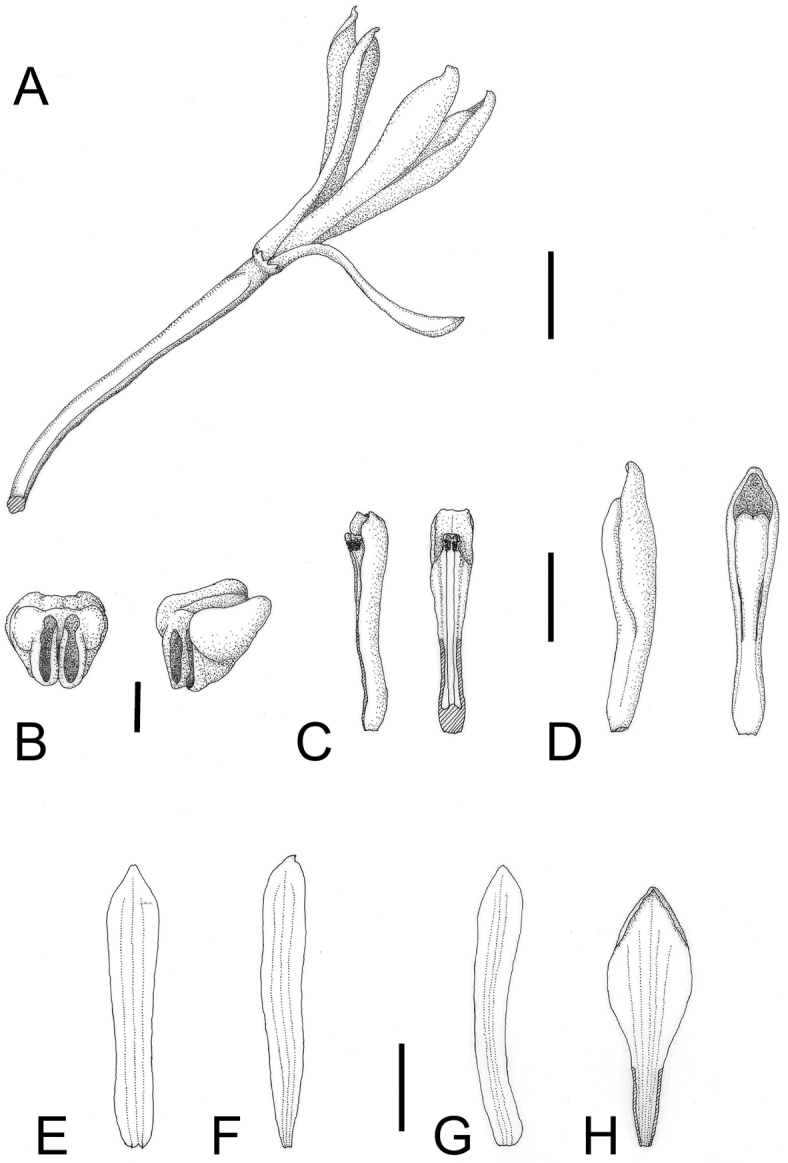
*Lecanorchis tabugawaensis*. **A** Flower **B** Anther cap **C** Column **D** Lip and column **E** Dorsal sepal **F** Lateral petal **G** Lateral sepal **H** Flattened lip. Bars: 5 mm (**A, C, D, E, F, G, H**); 1 mm (**B**). Line drawings by Kumi Hamasaki.

#### Conservation.

**IUCN red list category**: Critically Endangered, [CR D1]. To date, the distribution of *Lecanorchis tabugawaensis* appears to be restricted to two locations, separated by ca. 1.5 kilometers, along the Tabu and Onna Rivers at an elevation of ca. 100-180 m on the lower slopes of Mt. Aiko on the eastern Yakushima Island. The two known locations are located in humid evergreen broadleaved forests dominated by *Castanopsis sieboldii* (Makino) Hatus. ex T.Yamaz. & Mashiba and *Distylium racemosum* Siebold & Zucc. *Lecanorchis tabugawaensis* flowers in mid-July to early-August, and each location consists of only dozens of flowering individuals. The population of *Lecanorchis tabugawaensis* contains less than 50 mature plants, and at present we are not aware of any other locality where this species persists. Therefore, *Lecanorchis tabugawaensis* is classified as CR under Criterion D1 (IUCN 2014).

Although the lowland humid evergreen forests flanking the rivers in Yakushima Island have previously been identified as hotspots for endemic plant species, only a small proportion of the area is currently under protection (Yahara et al. 1987). This is in spite of the fact that 61% (20989 ha) of Yakushima Island is designated as a National Park, and 21% (10747 ha) is a World Natural Heritage site (Yahara and Tsukaya 2008). Consequently, one of the two locations of *Lecanorchis tabugawaensis* remains unprotected in an evergreen forest along the Tabu River adjacent to a *Cryptomeria japonica* (L.f.) plantation that has recently been cut. The area also harbors rare mycoheterotrophic plants, such as *Gastrodia uraiensis* T.C.Hsu & C.M.Kuo, *Gastrodia takeshimensis* Suetsugu, and *Gastrodia albida* T.C.Hsu & C.M.Kuo. Considering these mycoheterotrophs are completely dependent on their unique host fungi (e.g. Suetsugu et al. 2014b), it is important to conserve the entire ecosystem of their surrounding habitat. Further regulations restricting forest logging and construction are therefore required to conserve the flora, fauna, and the numerous endemic species restricted to low altitude habitats on Yakushima Island.

#### Etymology.

The specific epithet is derived from“Tabugawa”, which is the Japanese name for the Tabu River, the type locality that also harbors other rare mycoheterotrophic plants.

#### Taxonomic notes.

*Lecanorchis tabugawaensis* is similar to *Lecanorchis nigricans* Honda and *Lecanorchis taiwaniana* S.S. Ying. *Lecanorchis taiwaniana* has often been treated as a synonym of *Lecanorchis nigricans* Honda, a species known to be found in Japan, China, and Taiwan (Su 2000; Chen et al. 2009; Govaerts et al. 2016). However, this is based on the ambiguity of the original description by Ying (1987). Suetsugu et al. (2016b) reported that *Lecanorchis taiwaniana* can be easily distinguished from *Lecanorchis nigricans* by a combination of several characters (Table 1), including the longer peduncles, the longer rachis, the longer internodes, the narrower sepals and petals, the slightly 3-lobed labellum, the bright brown ascending capsules, the column that is more than half-fused with the labellum, the pubescence at the base of the column and the paler rachis coloration, based on the knowledge obtained by the newly discovered specimens. In addition, the differences of *Lecanorchis taiwaniana* (junior synonym: *Lecanorchis amethystea*) and *Lecanorchis nigricans* have been clearly stated by not only Suetsugu et al. (2016b) but also Sawa et al. (2006), Hsu and Chung (2010) and Lin et al. (2016).

**Table 1.** Morphological comparison between *Lecanorchis tabugawaensis* and its related species.

Characters	*Lecanorchis tabugawaensis*	*Lecanorchis taiwaniana*	*Lecanorchis nigricans*
Plant height	15–45 cm	15–45 cm	9–27 cm
Rachis color in developing stage	yellowish white	yellowish white	purplish white
Rachis color in fruiting stage	brownish black	brownish black	black
Rachis length	6–15 cm	(2–)6–15 cm	3–8 cm
Internode length of upper half of rachis	5–15 mm	5–15 mm	1–3 mm
Flower number	4–15	4–20	3–12
Sepal and petal color	yellowish white tinged with light purple	yellowish white tinged with light purple	purplish white
Width of sepal and lateral petal	2.0–2.5 mm	2.0–2.5(–3.0) mm	3.0–3.8 mm
Labellum shape	almost entire	indistinctly 3-lobed	almost entire
Colored area in labellum	ca. apical more than 2/3	ca. apical 1/4–1/5	ca. apical 1/3
Propotion of the columun fusion with labellum	2/5–1/2	3/5–2/3	ca. 1/2
Apical part of the adaxial labellum surface	glabrous	puberulent	puberulent
Pubescence at basal part of column	none	interspersed	none
Capsule color	bright brown	bright brown	black
Angle between capsule and inflorescence axis	20–45°	20–45°	70–90°

Data of the related species from Suetsugu et al. (2016b)

When comparing *Lecanorchis tabugawaensis* to *Lecanorchis nigricans*, *Lecanorchis tabugawaensis* has the taller inflorescences; the longer and lighter colored rachis; the yellowish-white, narrower sepals and petals; and the brighter brown suberect capsules. These characteristics of *Lecanorchis tabugawaensis* are shared with *Lecanorchis taiwaniana* (Table 1). However, *Lecanorchis tabugawaensis* can be distinguished from *Lecanorchis taiwaniana* by column morphology (straight vs. slightly curved), the pubescence at the base of the column (none vs. interspersed), labellum morphology (almost entire vs. 3-lobed), the width of the labellum when flattened (ca.5 mm vs. 6–7 mm), the proportion of the column fused with the labellum (2/5~1/2 vs. 1/2~2/3), the colored area of the labellum (2/3~1 vs. 1/4~1/5), and the apical part of the adaxial labellum surface (glabrous vs. puberulent for *Lecanorchis tabugawaensis* and *Lecanorchis taiwaniana*, respectively; see also Table 1).

#### Ecology.

Investigation on the column morphology suggested that the rostellum of *Lecanorchis tabugawaensis* is not very developed, as it does not function as a physical barrier between the stigma and the pollinia. As such, columns excised from flowers about one day after anthesis exhibit contact between the pollinia and the stigma because the pollinia begins to drop downward onto the stigma from the clinandrium. However, autonomous self-pollination in a bud stage is unlikely to occur because columns from the buds picked about a day before flower opening showed that pollinia are usually compacted within the clinandrium and basally inserted behind the apex of the stigma.

Autonomous self-pollination in Orchidaceae has previously been reported in various species, including the Vanilloideae subfamily, under which *Lecanorchis* belongs (e.g. Suetsugu 2013b, 2015b). Autonomous self-pollination has been proposed as an evolutionary response to ensure reproductive success given a lack of pollinators when the frequency of pollination is regularly quite low (Baker 1955). Mycoheterotrophic plants are often found growing on the dense forest floor, shaded by woodland or scrub. It can be theorized that mycoheterotrophy developed as an adaptation for these species to survive in such low-light conditions (Bidartondo et al. 2004). At the same time, pollinators may not be particularly suited to such low-light environments (Herrera 1995, 1997), thus indirectly creating a problem for plant reproduction if pollinators are unlikely to visit these areas. It appears that most of the mycoheterotrophic species investigated to date (especially nectarless species) have indeed abandoned an insect-mediated pollination system in favor of self-pollination (e.g. Suetsugu 2013a, b; Suetsugu 2014a; Suetsugu 2015c). Thus, autogamy in *Lecanorchis tabugawaensis* can also be considered a reproductive assurance to compensate for pollinator limitation due to their lack of nectar and pollinators’ habitat preferences.

## Supplementary Material

XML Treatment for Lecanorchis tabugawaensis
